# Exploring the potential application of alternative nuclei in NMR based metabolomics

**DOI:** 10.1007/s11306-023-02003-z

**Published:** 2023-04-15

**Authors:** Georgia M. Sinclair, Sophie M. Oakes, Andrew C. Warden, Amy M. Paten, Oliver A. H. Jones

**Affiliations:** 1 Australian Centre for Research on Separation Science (ACROSS), School of Science, RMIT University, Bundoora West Campus, PO Box 71, Bundoora, VIC 3083 Australia; 2 CSIRO Environment, Commonwealth Scientific and Industrial Research Organization (CSIRO), Research and Innovation Park, Acton, Canberra, ACT 2600 Australia

**Keywords:** Nuclear magnetic resonance spectroscopy, Metabolomics, Honey bees, ^13^C, ^15^N, ^31^P

## Abstract

**Introduction:**

Nuclear magnetic resonance (NMR) is widely used in metabolomics but it focusses on ^1^H over other NMR-active nuclei.

**Objectives:**

To evaluate the potential of alternative NMR-sensitive nuclei to generate useful metabolomic data.

**Method:**

Proton, carbon, phosphorus, and nitrogen-based NMR metabolomics was undertaken on extracts from mint and European honey bee tissue.

**Results:**

Carbon NMR provided useful information but required larger sample sizes. Phosphorus produced overlapping peaks in one dimensional (1D) analysis but showed potential in 2D experiments. ^15^N NMR was found to not be sensitive enough for general metabolomic work.

**Conclusions:**

Alternative NMR active nuclei are useful for metabolomics.

**Supplementary Information:**

The online version contains supplementary material available at 10.1007/s11306-023-02003-z.

## Introduction

Nuclear magnetic resonance (NMR) is a highly reproducible, non-destructive technique widely used in biochemistry due to its speed, robustness, ease of general use and minimal sample preparation requirements (Jones & Cheung 2007). NMR is also unmatched as a method for determining the structures of unidentified compounds. These features allow a wide range of small molecule metabolites to be identified and quantified across a large dynamic range. However, despite technical advances such as cryoprobes, new pulse sequences and ever more powerful magnets, ^1^H NMR metabolomics has not changed significantly in the last 20 years and its inherent lack of sensitivity has led to the field moving towards high-end mass spectrometry for metabolite analysis studies (Jones 2018). While extremely high-field (1.2 GHz) instruments coupled with Dynamic Nuclear Polarization (DNP) can potentially increase NMR sensitivity close to that of mass spectrometry, in practice this is difficult to accomplish (Jones 2018). However, the use of DNP in metabolomics-like studies has been reported (Dey et al. 2020).

The proton (^1^H) is the primary nucleus used in magnetic resonance-based metabolomics, not only because it provides the strongest signal of all the NMR-visible atomic nuclei (99.98% abundance) but also since it is ubiquitous in biological molecules. This means information-rich spectra can be obtained relatively easily. The proton is, however, not the only NMR-visible nucleus and, in the last 5–6 years, there has been an increase in efforts to determine whether alternative, NMR-active, biologically significant, nuclei, such as ^13^C, ^31^P and ^15^N, could provide useful information for metabolomics - without the need for isotope labelling (Markley et al. 2017; Bhinderwala et al. 2018; Jones 2018; Bhinderwala et al. 2020).

While far less NMR-sensitive than ^1^H, ^13^C is widely distributed in organic compounds. As such, it is well used in biological NMR in general, but not in metabolomics. The sensitivity of carbon NMR is limited as ^13^C is generally only 1% of total carbon (the other 99% being NMR-invisible ^12^C). Nevertheless, useful ^13^C based metabolomics is possible. For example, Wei et al. (2012a) used this method to successfully distinguish the species and origins of green coffee bean samples of *Coffea* *arabica* and *C. canephora* (robusta) from six different geographic regions, as well as to differentiate different degrees of coffee roast (Wei et al. 2012b). ^13^C NMR has also been shown to provide valuable insight into the positional distribution of fatty acids on the glycerol moiety and the stereochemistry of unsaturation in the metabolic analysis of olive oils (Lioupi et al. 2020) and distinguish between two different groups of tomato extracts (Dey et al. 2020).

Phosphorus is also well-distributed in biological systems. It plays a role in several important processes, including energy metabolism and nucleic acid synthesis. ^31^P NMR gives a good signal-to-noise (S/N) ratio, having essentially 100% natural abundance. It has been used in a number of areas, including in a study of the toxicity of the herbicide dinoseb (6-s-butyl-2,4-dinitrophenol) to fish embryos (Viant et al. 2006). The potential of ^31^P in metabolomics was the subject of an excellent recent review (Bhinderwala et al. 2020). ^31^P NMR generally yields sharp peaks and has a wide chemical shift range, and thus has featured in some metabolism studies, including investigations of the metabolic response of congestive heart failure (Michael O’Donnell et al. 1998), hypothermia (Liu et al. 2011) and toxicology (Viant et al. 2006). A combination of ^31^P NMR and chemical derivatization was also previously used to profile a number of important lipids in human serum (DeSilva et al. 2009).

Nitrogen-containing metabolites, such as adenine, biotin, and thiamine, are also common in cellular metabolism. The ^15^N nucleus is of low natural abundance (0.4% of total N). While ^15^N isotope labelling has been extensively used in NMR protein structure determination it is rarely used in metabolomics. The potential of tracer experiments with ^15^N- and ^13^C-labelled metabolites was recently reported (Bhinderwala et al. 2018). These studies showed the approach was able to simultaneously detect both ^15^N- and ^13^C-labeled metabolites in metabolic samples from *Escherichia coli* and *Staphylococcus aureus*.

Fluorine (^19^F) has a high gyromagnetic ratio, making ^19^F NMR measurements comparable with ^1^H NMR. Natural fluorinated metabolites do exist, the most common example is fluoroacetate which is used by various plant species worldwide as a defense mechanism against grazing by herbivores (Leong et al. 2017). However, as such compounds are rare they are not discussed further here.

For all nuclei, additional scans can be made to increase the signal-to-noise (S/N) ratio, which is particularly useful for the lower relative-abundance nuclei. However, there is a trade-off with acquisition time; signal varies directly with the square root of the number of scans. For example, if 4 scans gives a S/N = 1, 16 scans will give a S/N = 2, and so on. It is also worth noting here that, typically, only ^1^H NMR is considered reliably quantitative, although it is quite possible to set up experiments that allow other nuclei to give more quantitative data.

The use of alternate NMR-active nuclei clearly has potential for expanding coverage of the metabolome. This approach is hindered by the fact that there are no standard protocols for their use and a lack of knowledge about the kind of information each nucleus can provide. In this paper, we compare spectra produced using ^1^H NMR to those produced using ^13^C; ^15^N; and ^31^P, using mint and European honey bee extracts. Honey bees were used as a case study given the current interest in them for environmental and toxicology metabolomics studies and their economic benefits in the agriculture sector (du Rand et al. 2017; Colin et al. 2019). Mint was used for comparison. We show the types of spectra that can be obtained, and investigate the optimum sample size needed for such analysis. The aim of this short communication paper is to act as a theoretical, proof-of-concept work that will stimulate discussion of the potential of alternate nuclei in NMR metabolomics and continue the progress made by others in this area (Markley et al. 2017; Bhinderwala et al. 2018; Jones 2018; Bhinderwala et al. 2020).

## Methods

### Sample collection

Individual, adult, forager European honey bees (*Apis mellifera*), were collected from established hives located at the CSIRO Crace site (Canberra, ACT, Australia) and sent on dry ice to RMIT University (Melbourne, Victoria, Australia) where analysis was undertaken. Mint (*Mentha* spp.) was purchased from a local supermarket. Individual whole bees and mint leaves were homogenised using liquid nitrogen and a mortar and pestle. Homogenised samples were stored at − 20 °C prior to use.

### Chemicals

Methanol, chloroform and D_2_O were analytical grade and obtained from Sigma Aldrich (Castle Hill, NSW, Australia). Milli-Q water (18.2 MΩ.cm) was obtained in house.

### Metabolite extraction

Metabolite extraction followed a modified version of a previously described method (Belle et al. 2002). Briefly, pulverised samples were weighed in centrifuge tubes. To ensure there was enough tissue suitable for analysis, sample sizes for the experiment ranged between 0.1 g to 0.8 g. The samples were then sonicated for 15 min in analytical grade methanol at a volume three times the mass of the sample (i.e., a 100 mg sample was suspended in 300 µL of methanol). A 3:2 mix of chloroform:milli-Q water (18.2 mΩ) was then added in the same proportion as the methanol and the samples vortexed thoroughly for 10–20 s. Samples were then centrifuged for 20 min at 4000 rpm to generate distinct organic and aqueous layers. The aqueous layer from each sample was collected into a glass vial and evaporated to dryness under N_2_ at room temperature. The samples were then resuspended in 600 µL of D_2_O before NMR analysis.

### NMR spectroscopic analysis

The one-dimensional (1D) ^1^H, ^31^P, ^15^N and ^13^C NMR spectra were measured at 300 MHz, using a Bruker Advance 300 MHz NMR Spectrometer (Preston VIC) with a multi-channel probe. The parameters used are listed in the supplementary information as Table S1. Data were then visualised in Topspin™ (Bruker, MA, USA).

### Multivariate analysis

Principal Component Analysis (PCA) models intrinsic variation within the dataset, and is a popular and well used method for reducing the dimensionality of metabolomics data (Jones & Cheung 2007). PCA was conducted  on the ^13^C NMR data using the online MetaboAnalyst Software (version 5.0) (Pang et al. 2021). The raw data was uploaded to the server as a csv file. It was then pareto scaled and normalised to sum (each value in a row divided by the total sum of the row and multiplied by 100) and PCA carried out. The result is shown in Fig. 2.

## Results and discussion

### ^1^H NMR

A ^1^H NMR spectrum of aqueous-phase metabolites from the bee (*Apis mellifera*) and mint (*Mentha Sp.*) was obtained in order to establish a baseline using the standard NMR methods to compare later results against. A ^1^H spectra of bee tissue extract is shown in the supplementary information (Figure S1). Unsurprisingly, a better signal to noise ratio was obtained with a larger sample size; 44 peaks were detected using 0.1 g of sample while 110 peaks were seen with a sample size of 0.8 g. Most metabolomics studies use ~ 0.1 g of sample by default (Jones & Cheung 2007). It is therefore possible that using a greater amount could provide more data, even using standard ^1^H NMR, but often this is not necessary.

### ^13^C NMR

**Fig. 1:**
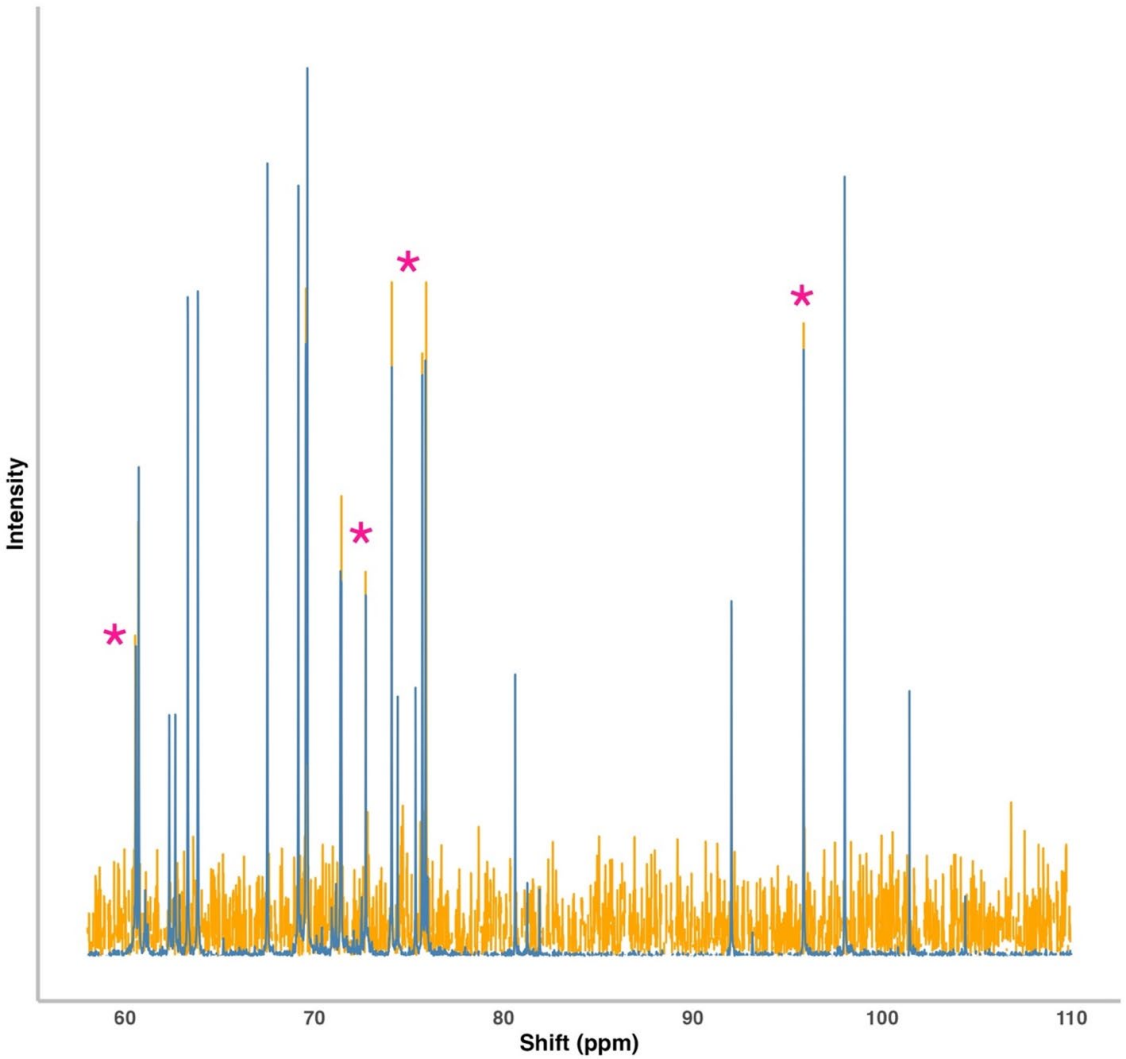
^13^C (decoupled) NMR spectrum of aqueous-phase metabolites from *Apis mellifera* in blue with a reference glucose spectrum in orange. Pink asterisks show locations of glucose peaks in the bee spectra

The same samples analysed with ^1^H NMR were then run via ^13^C NMR (Fig. 1). As ^13^C gives a much weaker signal than ^1^H, a much larger amount of sample (1 g rather than 0.1 g) is typically required to get a good signal-to-noise ratio. Interestingly, clearly identifiable peaks can still be seen using this method, even using a sample size of only 0.1 g. An example of this with Glucose is shown in Fig. 1.

Repeating the experiment using greater amounts of tissue in the extraction process resulted in a clear increase in signal strength (supplementary information, Figure S2). A total of 36 peaks were found in a decoupled ^13^C spectra (^1^H nuclei in the sample are broadly irradiated to fully decouple them from the ^13^C nuclei being analyzed) from 0.1 g of tissue compared to 58 using 1 g of tissue in the extraction.

**Fig. 2:**
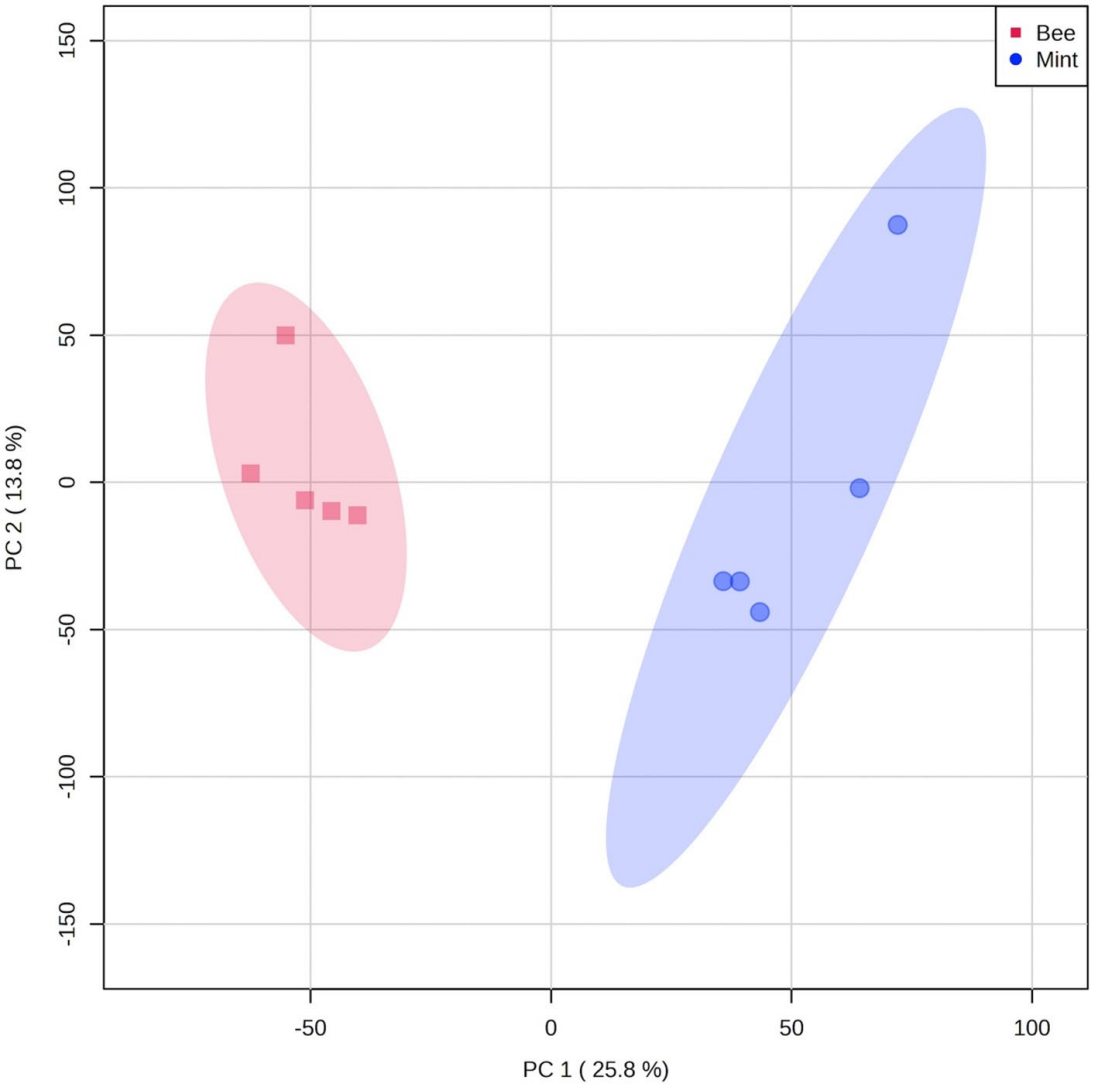
Principal Component Analysis scores plot of the ^13^C spectra from bee and mint samples

It also proved possible to differentiate the samples based on Principle Component Analysis (PCA) of the ^13^C spectra from bee and mint. This is shown in Fig. 2. This is consistent with the results reported by Dey et al. (2020), with the exception that we did not use DNP.

### ^31^P NMR

Although it has a wide shift range, the ^31^P signals from many phosphorylated compounds overlap. An example of this can be seen in the supplementary information. Figure S3 shows ^31^P NMR spectra from honey bee extract using two different sample sizes and features only two large peaks. These were tentatively identified as glycerophosphocholine which is used to store choline in the cytosol, and/or phosphatidylinositol, a key membrane constituent and signaling molecule. The lack of peaks meant that multivariate analysis would not have shown any useful information.

Despite the overlap of signals, ^31^P NMR has potential for characterizing biological samples and expanding the coverage of the metabolome either by 2D NMR or with library matching. For example, Bhinderwala et al., (2020), recently optimised a protocol to provide efficient integration of ^31^P NMR and enable the detection of metabolites used in critical cellular processes that would have previously been lost to peak overlap.

### ^15^N NMR

All samples were also run via ^15^N NMR*.* Due to its low natural abundance we did not expect to see any peaks using ^15^N without enrichment, and indeed this proved to be the case, even using sample masses of 1 g or higher (see supplementary information, Figure S4). This result was perhaps not unexpected, but we felt it important to confirm the data. Indeed, in science, one cannot assume results are “obvious”. Since there was no analyte signal there was no point undertaking multivariate analysis on this data set as it would have involved modelling nothing but noise.

Expanding coverage of the metabolome with nitrogen-based NMR is likely to require ^15^N-labeled metabolite tracer experiments (DeSilva et al., 2009). Bhinderwala et al. (2018) recently created a database consisting of 2D ^1^H–^15^N HSQC natural-abundance spectra of 50 nitrogen-containing metabolites to help with these forms of experiments. They also demonstrated the potential usefulness of the technique by labelling *Escherichia coli* and *Staphylococcus aureus* metabolomes. This work highlights the potential use of ^15^N for metabolomic studies but also indicates that specialised setups using isotopically labelled metabolites are needed for these types of experiments - which was not the aim of this work.

### 2D NMR

Most metabolomics studies use one dimensional spectra and utilise peak shape and shift value to assign peaks, in conjunction with the spectra of standard compounds. It is however hard to assign peaks in NMR of complex mixtures where most compounds have multiple peaks that overlap. One way around this is to use two-dimensional nuclear magnetic resonance spectroscopy (2D NMR) methods which give data plotted in a space defined by two frequency axes rather than one. 2D NMR is well known to organic chemists but often overlooked by metabolomic scientists who are often not NMR experts. The major advantage of 2D NMR over 1D NMR is the ability to distinguish between the overlapping signals. 2D Correlation spectroscopy (COSY) NMR experiments give confirmation of which protons are close to each other for example. Other experiments such as Heteronuclear Single Quantum Coherence (HSQC) spectroscopy provide information such as which protons are attached to which carbon. This can also be extended to nuclei such as ^31^P and ^15^N, although the spectra can be quite complex and require specialised knowledge to interpret. An example of a HSQC is shown in the supplementary information as figure S5. In this case the axes are the ^1^H-^13^C but other NMR active nuclei can also be used to help identify peaks of interest. Concomitant use of different NMR active nuclei, according to their natural abundance, could be an integrative approach to detect and monitor selectively different classes of chemical compounds. However, many metabolomic scientists are not aware of the increased resolving power offered by multidimensional NMR such as HSQC, COrrelation SpectroscopY (COSY), TOtal Correlation SpectroscopY (TOCSY), and so forth (Emwas et al. 2019).

### Sample size

Interest in using alternative nuclei in NMR metabolomics has grown in recent years, however, there is limited information regarding the optimum sample size required. To investigate this, we counted the number of peaks in samples extracted from differing amounts of honey bee tissue using the TopSpin™ software package. This data is shown in the Supplementary information (Table S2).

Increasing sample size increased the signal to noise value for all samples tested and also increased the number of peaks seen by ^13^C and ^1^H NMR. The number of peaks observed using ^31^P NMR did not increase with sample size and no signal was observed using ^15^N. These results indicate that alternative NMR nuclei may provide useful information even using standard sample sizes and extraction protocols and that large sample sizes are not always needed. Nitrogen NMR-based metabolomics will require either ^15^N labelled metabolites (Bhinderwala et al. 2018) or the use of hyperpolarization methods such as Spin Exchange Optical Pumping (SEOP) or para-enriched hydrogen through Signal Amplification by Reversible Exchange (SABRE) to generate enough signal to be useful (Jones 2018).

### Isotope enrichment

Isotope-based methods are commonly used for metabolic flux analysis in metabolomics. They are also commonly used in in protein NMR to increase the amount of a particular NMRactive nuclei. It entails replacing the nuclei of a certain NMR insensitive element with its respective isotope that is NMR active. For example, ^13^ C is naturally only ~ 1% of total carbon with the rest being NMR inactive ^12^C. By feeding an organism with C^13^ labelled substrate one can increase the percentage of this isotope and so increase the NMR signal it creates. This works well in microbial systems but is more difficult to do on larger animals because of the amount of labelled substrate needed to make an appreciable difference in the signal. Isotopic enrichment is not simple to use in metabolomics due to complications related to signal overlap. As noted by Lewis et al. (2010) the NMR spectra of unenriched biological extracts can contain thousands of ^1^H resonances. Spectra of ^13^C-enriched extracts are further complicated by ^1^H-^13^C J-couplings and complex mixtures of both ^13^C-labeled and unlabeled metabolites often result in NMR spectra with too many signal overlaps to give useful data. There are ways around this such as Isotope-edited TOtal Correlation SpectroscopY (ITOCSY) but as the results of this paper and Wei et al. (2012a, b) show, it is possible to useful ^13^C data for metabolomics without using isotype enrichment.

## Conclusions

The present study established and compared the additional information that can be produced with alternative NMR nuclei compared to standard proton-based NMR. The results highlighted that ^31^P and ^13^C, have potential in explorative metabolomic research. Approaches such as isotope labelling and hyperpolarisation can be investigated in future to increase the potential of ^31^P and ^15^N NMR based data. Sample sizes of 0.1 g provided sufficient data for good quality spectra using all nuclei except ^15^N, demonstrating that NMR approaches using alternative nuclei are not necessarily limited by the sample sizes generally used in NMR based metabolomics. The work highlights the potential use of alternative nuclei-NMR based metabolomics as a tool to aid in expanding and understanding the complex biochemical interactions occurring within individuals and contributes to the development of analytical tools in metabolomic studies.

## Supplementary Information

Below is the link to the electronic supplementary material.

Supplementary file1 (DOCX 421 KB)

## Data Availability

Raw NMR data were generated at RMIT University. All data are available from the contact author on request.
