# Supplementary material for: Exploring the potential application of alternative nuclei in NMR based metabolomics
Source: Metabolomics. 2023 Apr 15;19(4):42. doi: 10.1007/s11306-023-02003-z (PMC10105680; doi:10.1007/s11306-023-02003-z)
Supplement: Supplementary file 1 — Supplementary file1 (DOCX 421 KB) [file 11306_2023_2003_MOESM1_ESM.docx]

Supplementary Information for

Exploring the Potential Application of Alternative Nuclei in NMR Based Metabolomics

Georgia M. Sinclair ^1^, Sophie Oakes ^1^, Andrew C. Warden ^3^, Amy M. Paten (0000-0003-0420-2155) ^3^ and Oliver A. H. Jones (0000-0002-4541-662X) ^1,^*

^1^ Australian Centre for Research on Separation Science (ACROSS), School of Science, RMIT University, Bundoora West Campus, PO Box 71, Bundoora, VIC 3083, Australia

^2^ Land and Water, Commonwealth Scientific and Industrial Research Organization (CSIRO), Research and Innovation Park, Acton, Canberra, ACT 2600, Australia

***** Correspondence: oliver.jones@rmit.edu.au, +61 (3) 9925 2632

**Table S1.** NMR Parameters.

| **Target nuclei** | **Number of Scans** | **Bruker Pulse Program** | **Additional Parameters** |
| --- | --- | --- | --- |
| ^1^H | 128 | noesypr | Always with water suppression |
| ^13^C | 2000 | zpg30 | Decoupled |
| ^31^P | 2000 | zpg30 | Decoupled |
| ^15^N | 2000 | ineptrd | Direct |


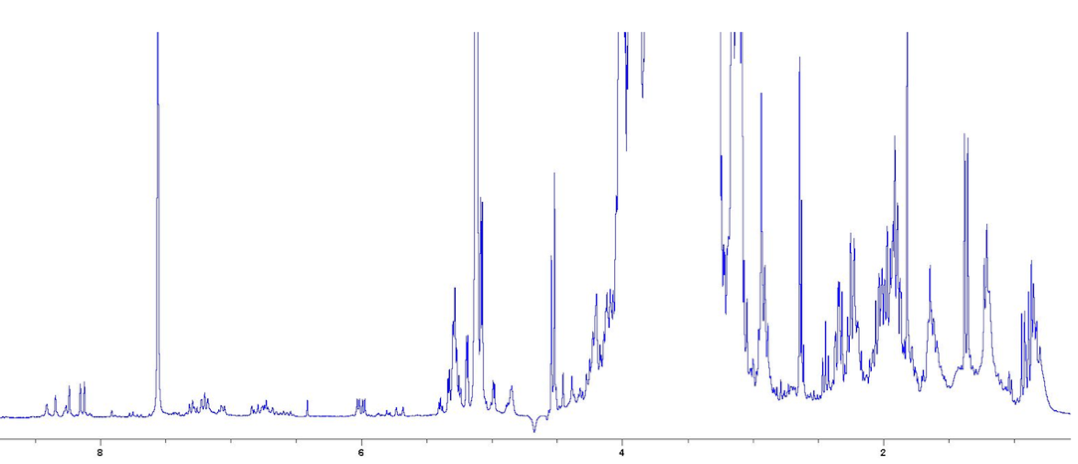


**Figure S1** ^1^H NMR spectrum of aqueous-phase metabolites from *Apis mellifera*. The x-axis is the chemical shift in ppm, the y-axis is peak intensity. Large numbers of peaks are visible.


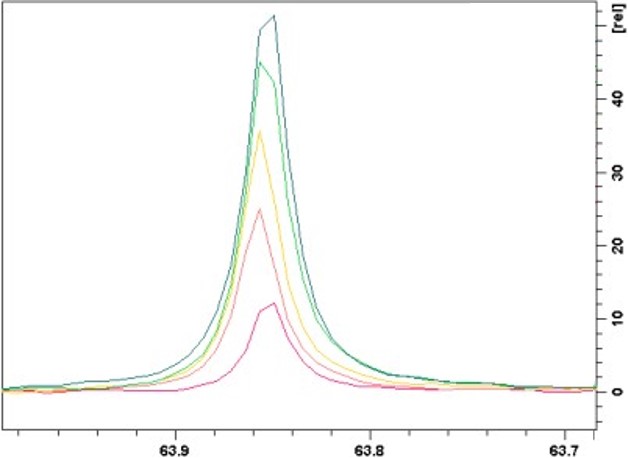


**Figure S2** ^13^C NMR spectra of aqueous-phase metabolites extracted from *Apis mellifera* tissue. The x-axis is the chemical shift in ppm, the y-axis is peak intensity. The sample size ranged from 0.1 g (red peak at the bottom) up to 0.8 g (blue peak at the top).


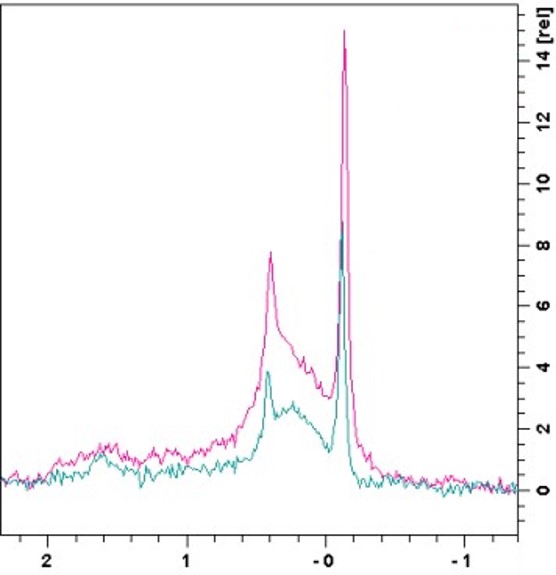


**Figure S3** ^31^P NMR spectrum of 0.1 g (blue) and 0.4 g (pink) of aqueous phase metabolites extracted from *Apis mellifera*. The x-axis is the chemical shift in ppm and the y-axis is peak intensity.


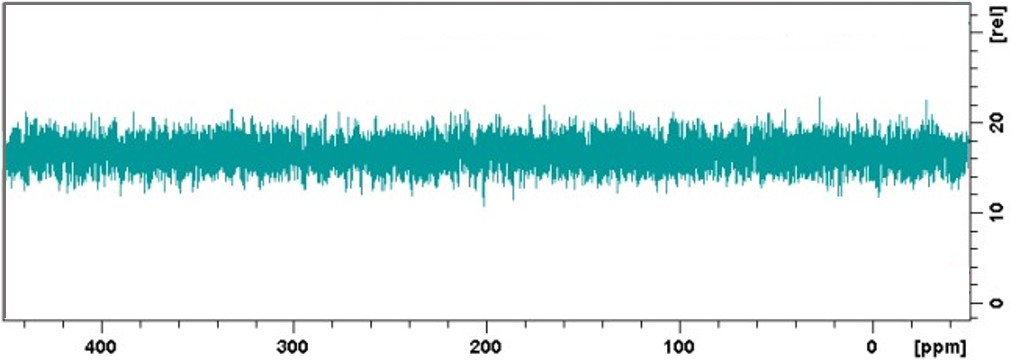


**Figure S4** ^15^N NMR spectrum of aqueous phase metabolites extracted from European honeybee (*Apis mellifera*). The x-axis is the chemical shift in ppm and the y-axis is peak intensity.


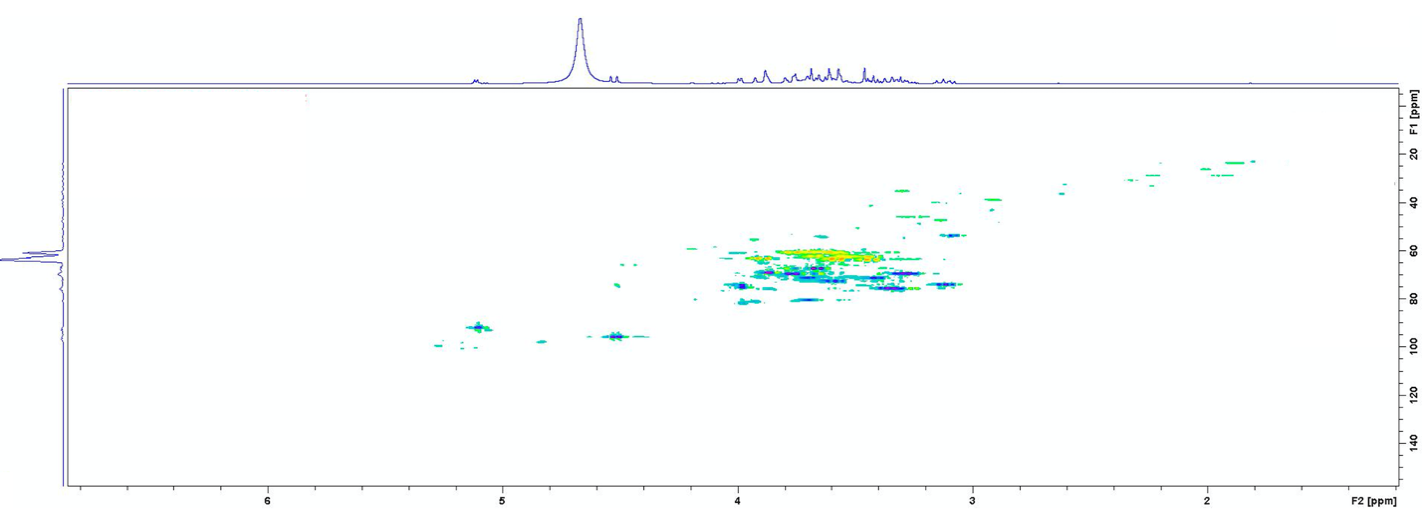


**Figure S5** ^1^H-^13^C Heteronuclear Single Quantum Coherence (HSQC) spectra of aqueous phase metabolites extracted from *Apis mellifera*. The x-axis is the ^1^H chemical shift in ppm and the y-axis is the ^13^C shift value. The peaks are colored by intensity (red = high intensity, blue/purple = medium intensity and green = low intensity.

**Table S2** The numbers of peaks identified using, ^1^H; ^13^C; ^31^P; with differing sample sizes.

| **Nuclei** | **Sample Size** | **Number of Peaks** | **Difference** |
| --- | --- | --- | --- |
| ^1^H | 0.1 g | 44 | +66 |
|  | 0.8 g | 110 |  |
| ^13^C | 0.1 g | 36 | +22 |
|  | 0.8 g | 58 |  |
| ^31^P | 0.1 g | 3 | 0 |
|  | 0.8 g | 3 |  |
| ^15^N | 0.1g | 0 |  |
|  | 0.8g | 0 | 0 |
